# Diagnostic challenges of primary central nervous system histiocytic sarcoma: case report and literature review

**DOI:** 10.3389/fonc.2025.1551157

**Published:** 2025-03-31

**Authors:** Na Liang, Changxian Chen, Dan Yuan, Qiang Xu, Yali Zhan, Yi Zhao, Di Wu, Cheng Yang, Chunming Li

**Affiliations:** ^1^ Department of Pathology, School of Basic Medicine, Zunyi Medical University, Zunyi, China; ^2^ Department of Pathology, Affiliated Hospital of Zunyi Medical University, Zunyi, China; ^3^ Department of Urology, the Second Affiliated Hospital of Zunyi Medical University, Zunyi, China; ^4^ The First Clinical College of Zunyi Medical University, Zunyi, China; ^5^ School of Forensic Medicine, Zunyi Medical University, Zunyi, China

**Keywords:** histiocytic sarcoma, primary central nervous system, diagnosis and differential diagnosis, case report, immunohistochemistry

## Abstract

Histiocytic sarcoma (HS) is a rare malignant tumor that primarily affects the lymph nodes, intestines, skin, and soft tissues. Primary central nervous system histiocytic sarcoma (PCNSHS) is even rarer. We present the case of a 50-year-old Asian male with PCNSHS who was hospitalized after experiencing intermittent headaches and slurred speech for a week. During surgery, both tumor cells and purulent material were observed, and the diagnosis of PCNSHS was ultimately confirmed by immunohistochemistry. Furthermore, we conducted a literature review from 1952 to the present, screening and analyzing 49 related cases across 41 publications.

## Introduction

Histiocytic sarcoma (HS) is a rare malignant tumor arising from the lymphohematopoietic system, commonly affecting the lymph nodes, intestines, skin and soft tissues (1). Primary central nervous system histiocytic sarcoma (PCNSHS) is extremely rare and can occur in the brain (2), cerebellum (3), leptomeninges (4) and spinal cord (5). According to the 2021 World Health Organization classification of central nervous system tumors, HS is classified as a histiocytic tumor (6). PCNSHS is often associated with a pronounced inflammatory response, such as lymphocyte infiltration, which may affect the diagnosis (7). Sometimes purulent material can also be found during surgery, which may be result from uncontrolled proliferation of monocytes or macrophages and can easily be misdiagnosed as an infectious abscess (3). The specific cause of PCNSHS has not yet been determined. However, studies suggest that PCNSHS may be associated with prior radiation therapy and complex cytogenetic abnormalities in the tumor (8). Currently, there is no standardized treatment plan for PCNSHS. Surgery is the main treatment, especially for patients with single lesions, and radiotherapy and chemotherapy are often used as adjuvant treatments (9). This study reports a 50-year-old Asian male patient with PCNSHS, focusing on the diagnosis and differential diagnosis of previously published cases. Through a comprehensive literature review and analysis, we aim to enhance clinicians’ diagnostic accuracy for PCNSHS, facilitating more effective identification and management of this condition.

## Case presentation

**Figure 1:**
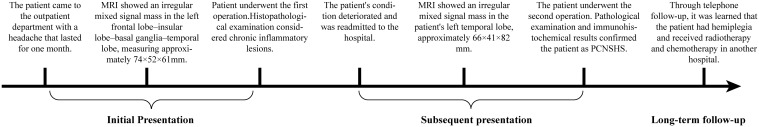
Timeline of the patient’s medical progress.

**Figure 2:**
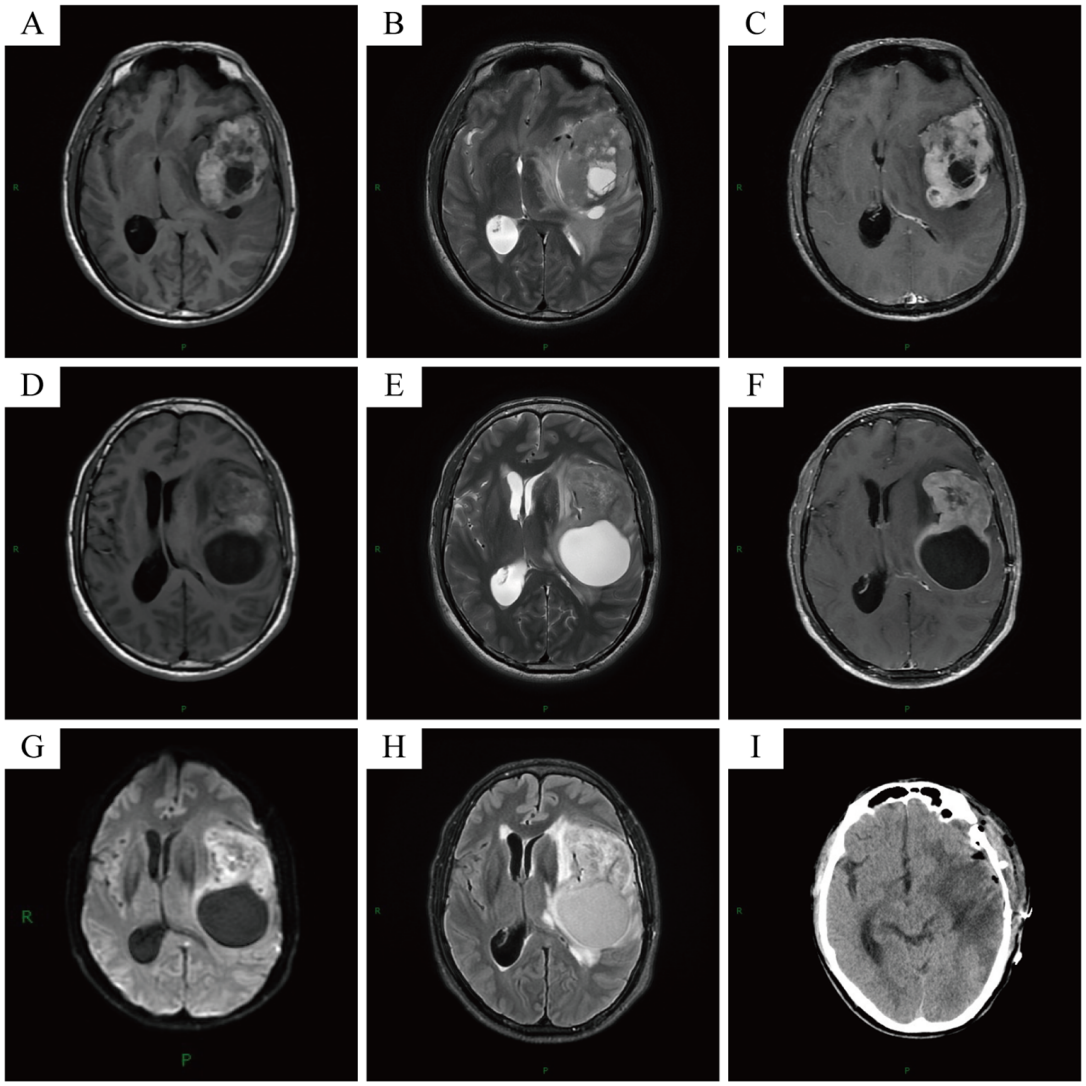
Initial MRI showed an irregular mixed signal mass in the patient’s left frontal lobe-insular lobe-basal ganglia region-temporal lobe, approximately 74×52×61mm in size **(A-C)**. Pre-operative MRI showed an irregular mixed signal mass in the patient’s left temporal lobe, approximately 66×41×82mm in size **(D-H)**. Post-operative CT showed post-operative changes in the patient’s left cerebral hemisphere **(I)**.

**Figure 3:**
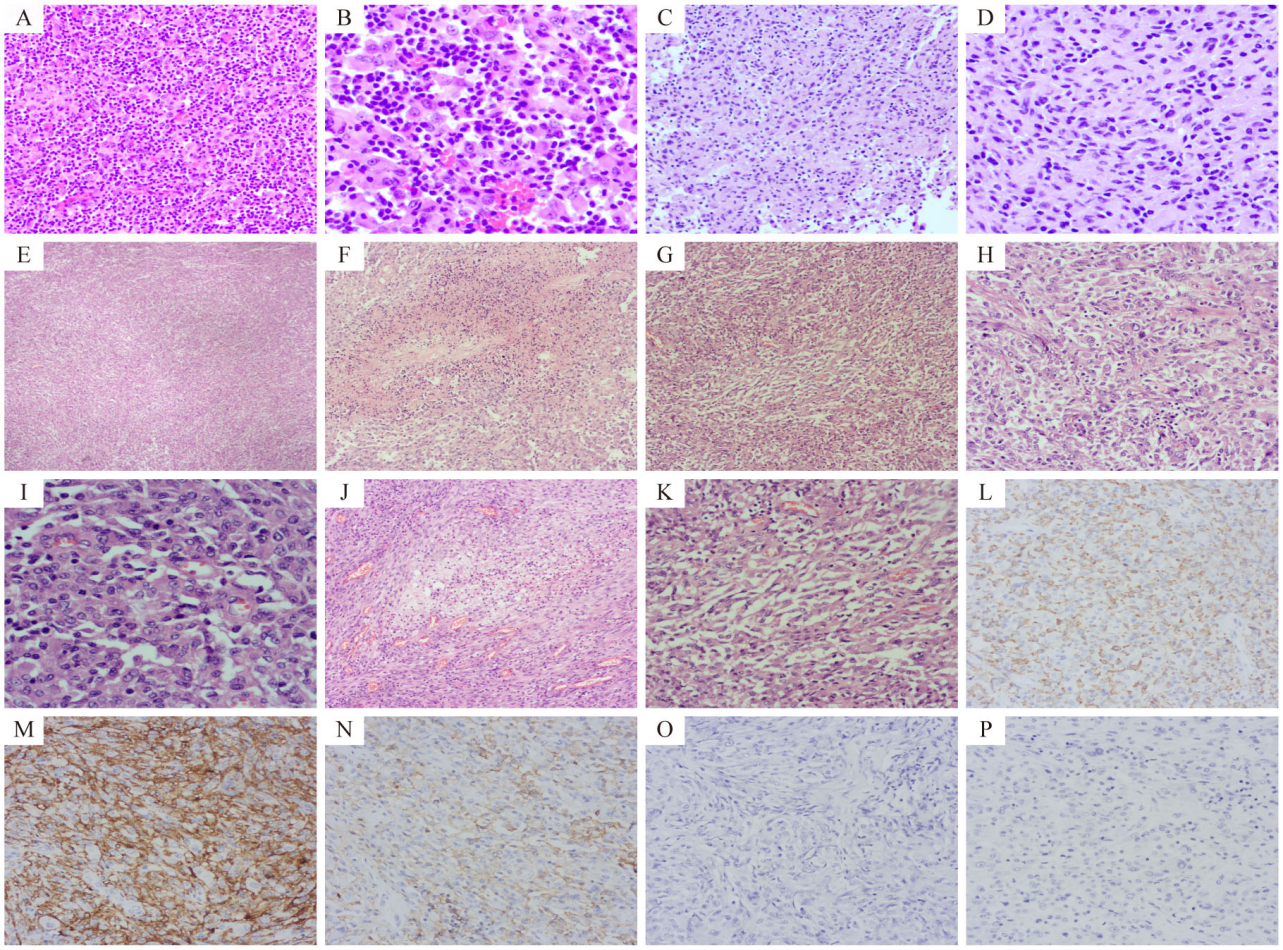
The results of hematoxylin-eosin (HE) staining and immunohistochemical (IHC) assays. Pathological sections after the first operation, showed a large number of lymphocytes and a small number of histiocytes (**A**, HE 200× and **B**, HE 400×). Frozen pathological sections during the second operation showed that the tumor cells were distributed in sheets and similar in shape to gemistocytes (**C**, HE 200× and **D**, HE 400×). Pathological examination after the second operation showed: Tumor cells were arranged in diffuse patches (**E**, HE 40×) with necrotic areas (**F**, HE 100×). Tumor cells appeared spindle-shaped (**G**, HE 100×) and epithelial-like (**H**, HE 200×). Tumor cells exhibited significant dysplasia and pleomorphism, and mitotic figures were common (**I**, HE 400×). Foam cells were seen in some areas (**J**, HE 100×), and a large number of inflammatory cell infiltrates were observed in some areas (**K**, HE 200×). Tumor cells were positive for CD68, CD163, and CD4 (**L–N** respectively, IHC 200×), and negative for GFAP and Oligo-2 (**O, P**, IHC 200×).

The Medical Progress Timeline (**Figure 1**) summarizes three key phases:

1. Initial Presentation (February 2024): A 50-year-old Asian male patient presented to our hospital’s outpatient department due to persistent headache for one month. Initial magnetic resonance imaging (MRI) showed an irregular mixed signal mass in the left frontal lobe–insular lobe–basal ganglia–temporal lobe, measuring approximately 74×52×61mm. T1WI showed an uneven high signal, and T2WI showed an uneven equal/slightly high signal. The enhanced scan showed obvious uneven enhancement of the tumor (**Figures 2A–C**). The patient underwent neuroendoscopic resection of ventricular lesions and neuronavigation resection of intracranial lesions. Histopathological examination showed that numerous lymphocytes and a small number of histiocytes with abundant cytoplasm, vacuolated nuclei, and pronounced nucleoli were seen in the excised tissue (**Figures 3A, B**). A chronic inflammatory lesion was initially considered.
2. Subsequent presentation (approximately 3 months after the initial presentation): After regular follow-up, the patient’s headache symptoms worsened again, accompanied by slurred speech for a week, and he was readmitted to the hospital. Preoperative MRI showed an irregular mixed-signal mass in the patient’s left temporal lobe, approximately 66×41×82 mm in size. It showed inhomogeneous high signal intensity on T1WI and inhomogeneous iso/slightly high signal intensity on T2WI. On enhanced scan, the solid part of the tumor showed obvious uneven enhancement, and DWI showed high signal intensity (**Figures 2D–H**). The patient underwent a second intracranial lesion resection. Intraoperatively, the patient’s left temporal lobe was found to be swollen with dura mater invasion, and about 30 mL of light yellow pus was drained from the tumor. The tumor measured approximately 5×5×6 cm. Intraoperative histopathology revealed that the tumor cells were arranged in sheets, had abundant cytoplasm, and exhibited eccentric nuclei, resembling gemistocytes (**Figures 3C, D**). The frozen section diagnosis was glioma. Postoperative computed tomography (CT) revealed a residual cavity, gas, and blood accumulation in the left temporal lobe, along with a large patchy hypodense area in the left cerebral hemisphere (**Figure 2I**). Finally, postoperative pathological examination was conducted (**Figures 3E–P**). Immunohistochemistry showed that the tumor cells were positive for molecules such as CD68 and CD163. After ruling out other negative markers and conducting an intra-departmental consultation, the patient was finally diagnosed with histiocytic sarcoma of the temporal lobe of the left lateral fissure cistern. The patient’s postoperative course was stable, with no complications, and he was discharged.
3. Long-term follow-up (approximately 12 months after the initial presentation): A follow-up call revealed that the patient’s tumor had recurred, and he had developed hemiplegia. The patient had received chemoradiotherapy at another hospital.

### Pathological findings

Histopathological examination of the second resection specimen showed diverse tumor cells with significant dysplasia, necrosis, mitotic figures, foam cells and inflammatory cell infiltration (**Figures 3E–K**).

Immunohistochemical staining of the second resection specimen showed that tumor cells were positive for CD68, CD163, CD4, INI-1 and ATRX (**Figures 3L–N**), Scattered focal positivity for lysozyme, and some positive for S-100 and LCA. The Ki-67 proliferation index was about 30%. The tumor cells were negative for CD34, MPO, CD117, CD43, CD21, GFAP, Oligo-2, IDH-1, EMA, PR, SSTR2, ALK, SMA, Desmin, H-caldesmon, MyoD1, TFE3, HMB45, Melan-A, and Langerin (**Figures 3O, P**). Based on histopathological and immunohistochemical results, PCNSHS was diagnosed.

### Systematic review of literature

By searching the Pubmed database for case reports on PCNSHS published between 1952 and 2024, a total of 191 results were obtained using the following keywords “((((nervous system) OR (neurological disorder)) OR (brain)) OR (neurological symptom)) AND (histiocytic sarcoma)”. After removing duplicates, excluding cases of metastatic central nervous system histiocytic sarcoma, and excluding animal-related studies, 49 cases reported in 41 articles were identified, excluding our own cases.

**Figure 4:**
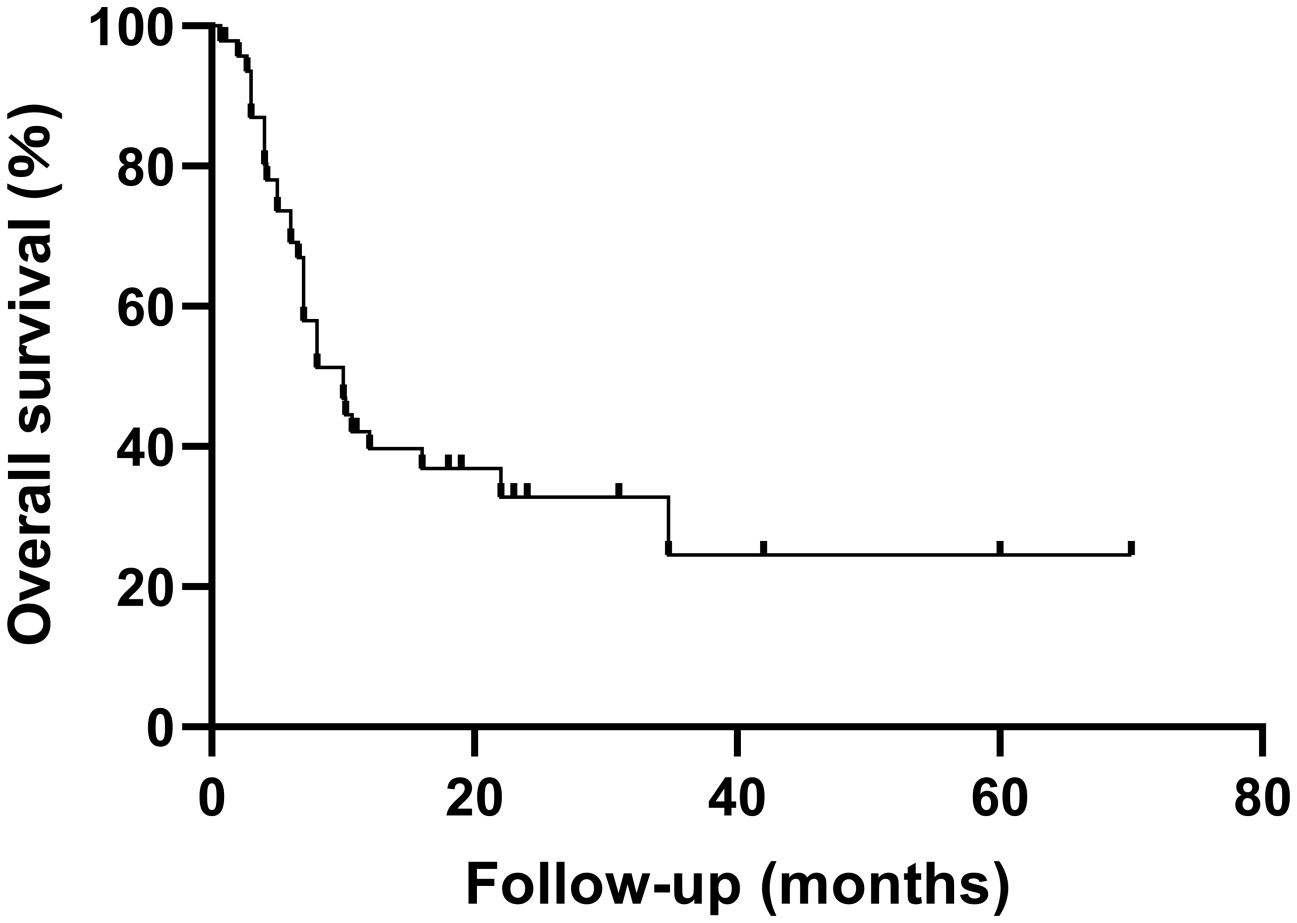
Kaplan-Meier survival curves for patients with PCNSHS.

A summary of previously reported cases of PCNSHS (**Table 1**, **Figure 4**) showed that a total of 49 patients were included (male to female ratio of 0.88), with no significant gender differences. PCNSHS can develop at any age, ranging in age from 17 months to 71 years, with a median age of 43 years at diagnosis. The mortality rate was 61%, and the median survival was 10 months, indicating a poor prognosis. Tumors can affect all parts of the central nervous system, with the most common sites being the frontal lobe (12.3%), corpus callosum (8.2%), parietal lobe (6.8%), pia mater (6.8%), and cerebellum (5.5%). Patients can present a variety of clinical symptoms, with headache (21.6%) being the most common, followed by vomiting (12.9%), dizziness (8.6%), nausea (7.8%), and gait disturbance (6.9%). It should be noted that the symptoms of some patients gradually worsen over weeks to months, suggesting that PCNSHS may be difficult to detect in the early stages and require high vigilance for progressively worsening neurological symptoms.

**Table 1:** Summary of previously published cases of primary central nervous system histiocytic sarcoma (PCNSHS).

Case No	Age/Sex	Tumor location	Size(cm)	Clinical presentation	Immunohistochemical markers	Treatment	Follow-up, months	Outcome	Reference
1	27/F	Right frontal lobe	NA	Shortness of breath, palpitations and fatigue	CD163(+),lysozyme(+),CD10(+),CD13(+),ALK(+)	CT	24	Alive	2024 (34)
2	6/M	Right frontal lobe	3.3 × 2.9 ×2.9	Headaches and nausea for 3 weeks	CD68(+),CD163(+),CD4(+),S100(+)	Surgery+RT	19	Alive	2024 (22)
3	59/M	Bilateral frontoparietal area	NA	Worsening headache, accompanied by nausea, vomiting, vision changes, difficulty balance	CD163(+),CD68(+),PU.1(+),CD14(+),CD4(+),fastin(+)	RT+CT	7	Died	2024 (22)
4	30/F	Right cerebellar	3.6 × 3.0	Progressive dizziness and headache	CD163(+),ATRX(+),H3K27ME3(+)	Surgery+RT	12	Alive	2024 (35)
5	9/F	Anterior falciform region	NA	5 days of head and neck pain,vomiting	CD34(+),CD68(+),CD163(+), S-100(+),Ki-67(25%),CD1a(−),Langerin(−),BRG-1(−),INI-1(−),SALL-4(−),BRAF V600E(−)	Surgery+RT	3	Alive	2024 (20)
6	41/M	Brain	NA	3 months of dizziness, gait difficulty, hearing loss	CD68(+),lysozyme(+),CD34(+),vimentin(+),CD3(−), CD20(−), CD1a(−), S-100(−),CD79a(−)	Surgery	10.7	Died	2023 (2)
7	36/F	Frontotemporal	NA	3 months of impaired vision	NA	Surgery+RT+CT	70.0	Alive	2023 (2)
8	42/M	Frontoparietal	NA	6 months of limb weakness	NA	Surgery	NA	NA	2023 (2)
9	27/M	Triangle area	NA	0.5 months of headache, nausea, vomiting	NA	Surgery+RT+CT	34.8	Died	2023 (2)
10	67/M	Occipital	NA	1 months of dizziness	NA	Surgery+RT+CT	10.2	Died	2023 (2)
11	40/F	Brain	NA	1 months of headache	NA	Biopsy	1	Alive	2023 (2)
12	24/F	Right parietal lobe region	2.9 × 3.0 × 2.3	Numbness of the left face and fingertips for 1 year	CD68 (+), CD163 partly (+), S-100(+), BRAF V600E(+),PD-L1(+),Ki-67(70%)	Surgery+RT+ apatinib + anlotinib	10	Died	2022 (11)
13	35/M	Right frontotemporal	4 × 7.7 × 4.6	Headache and swelling of the right parietal bone and orbit	CD68 (+), CD163 (+),Ki-67(diffusely positive expression)	Surgery+RT	12	Alive	2022 (36)
14	50/F	Periventricular	NA	Right facial pain and numbness and paresthesia of the right first through third fingers	CD163(+), CD68(+), lysozyme(+)	Immunotherapy	16	Died	2022 (37)
15	31/F	Right medial temporal lobe	NA	2 month of diplopia, headache, seizures, and vomiting	CD68 (+), lysozyme (+), CD163 (+), vimentin (+), S100 (+),ALK-1(−), BRAF V600E (−)	Surgery+RT	6	Died	2022 (38)
16	66/M	Right frontal lobe, right corona radiate, corpus callosum, and left periventricular region	NA	Progressive bladder incontinence, lower limb weakness, and cognitive decline over 2 month duration	CD68 (+), lysozyme (+), CD163 (+), vimentin (+), S100 (+),ALK-1(−), BRAF V600E (−)	Surgery	5	Died	2022 (38)
17	56/M	Skull base	2.3×1.2 × 0.3	7 weeks of right-sided headache	CD4(+),CD11c(+),CD14(+),CD45(+),CD163(+), CD68 (+), PD-L1(−)	Surgery+RT	3	Died	2022 (18)
18	42/F	Diffuse leptomeningeal lesions	NA	1 month of intermittent headaches and vomiting	CD163(+),CD3(−),CD20(−), PAX-5(−),CD45(−), CKAE1/AE3(−)	No therapy	3	Died	2022 (4)
19	67/M	Periventricular	0.1~1.4	Progressive behavioral disturbances, cognitive decline, progressive ataxia and headaches of 3 months	CD68(+),CD163(+),Ki-67(30%), BRAF V600E(−),PTEN(−)	No therapy	7	Died	2019 (24)
20	65/F	Left frontal lobe	6.3	Impaired orientation	CD68(+),CD163(+),CD45(+), CD4(+),ALK(−),PD-1(−), PD-L1(−)	Surgery+RT	22	Died	2018 (39)
21	47/F	Cerebellar, leptomeninges	2.9 × 3.0 × 2.3	Progressive ataxia, headaches, imbalance, nausea, vomiting, and diplopia	CD163(+),CD68(+),CD45(+),Vimentin(+),PD-L1(+),PD-L2 (+)	Surgery+RT+CT	8	Died	2018 (9)
22	49/F	Parietal lobe	2.2 ~ 2.5	2 months of hypomnesia, odynophagia, gait disorder	CD45(+),lysozyme(+),CD68 (+),CD163(+),Ki-67(60%)	Surgery+RT	8	Died	2018 (12)
23	45/F	Leptomeninges of the brain and spinal cord	NA	10 years history of pituitary adenoma; 1 month headache, vomiting, unsteadiness	CD68(+),CD163(+), Lysozyme(+),CD4(+),CD45 (+),BRAF V600E(−), ALK-1(−)	No therapy	2	Died	2017 (1)
24	16/M	Corpus callosum, lateral ventricle	6.5 × 5.3	Dizziness, left lower limb weakness and limping gait for 2 months	CD163(+),CD68(+),lysozyme (+),CD4(+),INI-1(+),BRG1 (+),Ki-67(30%)	Surgery+RT+CT	12	Died	2017 (40)
25	47/F	Cerebellum	NA	Increasing difficulty with ambulation, speech, and energy level over several weeks	CD68(+),CD163(+),CD45(+), vimentin(+),CD43(+)	Surgery+RT+CT	NA	NA	2017 (3)
26	65/M	Frontal and parietal lobe of masses, spinal cord and meningeal dissemination	NA	3 months of progressive impaired consciousness and headache	CD68(+),CD163(+),α1-antichymotrypsin(+),α1-antitrypsin(+),CD4(+),LCA(+),S-100(+), lysozyme(+)	RT	11	Alive	2016 (13)
27	51/F	Subarachnoid space, cavernous sinus, pituitary, sella, ependyma, brain parenchyma, corpus callosum, optic tracts, leptomeninges, and the T3-7 epidural spaces	NA	Progressive memory loss, blurred vision, lower extremity weakness, back pain, and constipation	CD68(+),CD163(+),CD1A(−),Langerin(−),S-100(−)	CT	7	Died	2016 (25)
28	59/M	Cerebral hemisphere, spinal cord	NA	Right side weakness and numbness in the left leg for 2 months	CD68(+),CD163(+),S-100(+)	CT	8	Died	2015 (5)
29	43/F	Periventricularly, parenchymal, pons	NA	6 months of progressive nocturnal nausea and vomiting	CD45(+),CD163(+),CD68(+),CD4(+),Ki-67(20%)	RT+CT	3	Died	2015 (41)
30	15/F	Right frontal lobe	5.8 × 4.7 × 4.0	3 months of worsening occipital headache, vomiting, diplopia, weight loss, and progressive lethargy	CD163(+),CD68(+),CD33(+), lysozyme(+),GFAP(−)	Surgery+RT+CT	23	Alive	2015 (32)
31	61/M	Left cavernous sinus/Meckel’s cave	1.5×1.1×1.8	Tongue paresthesia	CD68(+),lysozyme(+),CD163 (+),CD3(+),CD5(+),CD8(+), S-100(+),Myeloperoxidase(+)	RT+CT	31	Alive	2015 (42)
32	23/M	Left cerebellopontine angle	6	Headache, left-sided diplopia, left facial numbness, and loss of smell and taste	CD68(+),CD4(+),lysozyme(+),CD163(+)	CT+RT	60	Alive	2015 (26)
33	63/F	Cranial nerve	NA	2 months of stabbing paraesthesia	CD68(+),lysozyme(+),CD163(+),iBa1(+),Mic99(+),vimentin(+),HLA-DR(+),Ki-67(24%), ALK(−)	CT	0.7	Died	2014 (43)
34	40/M	Left temporal lobe	NA	Memory impairment, paroxysmal right-sided hemibody paresthesia, and neck pain	CD68(+),CD4(+),CD163(+), S100(+)	Vemurafenib	6	Died	2014 (33)
35	52/M	Frontal lobe	NA	15 days of weakness of left lower limb	CD68(+),lysozyme(+),S-100(+),vimentin(+)	Surgery+RT+CT	16	Alive	2014 (7)
36	50/M	Right parieto occipital	1.7	The pineal gland of recurrent cavernous hemangioma follow-up	CD163(+),CD68(+),CD4(+), fascin(+),Ki-67(15~20%),ALK (−)	Surgery+RT	18	Alive	2013 (8)
37	41/F	Temporal lobe	1.5 × 2	Headache, generalized weakness, and chills	CD68(+),CD163(+),lysozyme(+),S100(+)	Surgery+RT+CT	42	Alive	2013 (44)
38	58/M	Bifrontal	6.5 × 5	3 weeks of gait instability and memory difficulties	CD163(+),CD68(+),LCA(+), α1-antitrypsin(+),CD4(+), lysozyme(+)	Surgery	4.2	Died	2013 (45)
39	44/M	Corpus callosum, right lateral ventricle	3.5;2.6	2 weeks of unsteadiness, loss of balance, and left-sided weakness	CD11c(+),CD68(+),CD 163(+)	RT+CT	6.6	Died	2013 (27)
40	16/M	Left parietal lobe	3.5 × 4.4 × 4.0	2 months of intermittent progressive headaches	CD68(+),CD45(+),CD45RO(+),CD15(+)	RT	4	Died	2013 (14)
41	55/F	Multiple lesions of brain parenchyma	NA	Hypomnesia and gait disorder lasting about 2 months	CD45(+),CD68(+),CD163(+),Vimentin(+),S-100(+)	Surgery+RT	4	Died	2012 (46)
42	17 months/F	Right hemisphere of the cerebellum	4.7 × 4.3 × 4.3	2 weeks of progressive gait disturbance	CD68(+),CD163(+),vimentin(+),	Surgery+CT	16	Alive	2012 (47)
43	38/F	Multiple cerebral supratentorial	5	1 ~2 months of headaches associated with nausea, vomiting and dizziness	CD45(+),CD68(+),CD163(+)	RT+CT	2.7	Died	2012 (19)
44	43/F	Multiple lesions of brain parenchyma and spinal cord	NA	Progressive ataxia, headache and altered general status lasting for 3 weeks	CD68(+),CD163(+),CD14(+), S-100(+)	CT	10	Died	2012 (48)
45	62/F	Meningeal solitary lesion of tentorium cerebelli	NA	1 month of general malaise, dizziness, unsteadiness, headache and vomiting	CD163(+),CD68(+),lysozyme(+)	Surgery	24	Alive	2012 (15)
46	34/M	Right frontal region	2	8 weeks of right-sided scalp numbness and tenderness, headaches	CD163(+),CD68(+),lysozyme(+)	Surgery	10	Alive	2012 (15)
47	71/F	Intramedullary	2.5 × 1 × 1.1	1 month of back pain and progressive right lower extremity weakness	CD163(+),CD68(+),CD45(+), lysozyme(+)	Surgery+RT	5	Died	2010 (49)
48	53/F	Right retroorbital	3.1 × 2.9 ×2.2	Visual impairment, unsteadiness, headache	CD163(+),CD68(+)	Surgery+RT	7	Died	2007 (50)
49	13/M	Left occipital lesion, meninges	1.1	3 months of headaches, seizures, shoulder pain	CD68(+),HAM56(+), lysozyme(+)	Surgery	4	Died	2003 (23)

F, female; M, male; NA, not available; CT, chemotherapy; RT, radiotherapy.

## Discussion

In 1970, Mathé et al. first reported HS, a malignant tumor originating from the lymphoid-hematopoietic system (10). HS is rare and typically occurs in lymph nodes, intestines, skin, and soft tissue. PCNSHS is even rarer and can occur in the brain, cerebellum, spinal cord, and leptomeninges (2–5). The histomorphology of PCNSHS is characterized by pleomorphic large cells, usually with abundant eosinophilic cytoplasm and irregular nuclei, accompanied by prominent nucleoli. Tumor cells proliferate actively, with common mitotic figures observed, and tumor necrosis may occur. In addition, tumor giant cells, red blood cell phagocytosis, or focal spindle cells can also be seen in some cases (1, 5, 11–13). PCNSHS is typically accompanied by significant inflammatory cell infiltration, including neutrophils, eosinophils, and plasma cells. This significant inflammatory response is an important feature that distinguishes PCNSHS from other forms of HS (14, 15). The histomorphology of this case was consistent with the histopathological results of previously published PCNSHS cases, exhibiting typical histological features and inflammatory reactions, further supporting the diagnosis of PCNSHS.

PCNSHS is a diagnosis of exclusion. Immunohistochemistry plays a crucial role in diagnosing PCNSHS by identifying the histiocytic lineage of tumor cells using specific markers. Diagnosis of HS (including PCNSHS) requires at least two positive markers, including CD68, CD163, CD4 and lysozyme, and negative markers are also needed to rule out other diseases (16). CD163 is a hemoglobin-clearance receptor that is expressed only on monocyte and histiocytic lineages. Compared to CD68, CD163 has stronger specificity in identifying histiocytic lineages and diagnosing HS (17). Interestingly, a study reported a case of PCNSHS with only weak and focal expression of CD163. In this regard, one hypothesis is that this case may show a similar phenotype to M1 macrophages, which typically have low CD163 expression; another hypothesis is that some HS cases may show partial loss of specific markers (18). In our case, CD4, CD68, and CD163 were all positive, and Lysozyme showed a small amount of sporadic positive expression, suggesting that the tumor cells originated from histiocytes.

The diagnosis of PCNSHS requires the systematic exclusion of tumors with overlapping morphological and molecular characteristics by integrating immunohistochemical results and molecular characteristics in multiple dimensions. Preliminary immunohistochemical assessment should focus on identifying histiocytes lineages, such as CD163, CD68 and other histiocytes markers. Secondly, tumors with overlapping morphological features must be excluded using ancillary techniques. For example: S-100 positivity is observed in melanoma, interdigitating dendritic cell sarcoma, and Rosai-Dorfman disease. However, S-100 is also expressed in approximately 33% of HS cases. Therefore, a positive S-100 does not rule out the diagnosis of HS (12). Negative EMA, CD34, SSTR2, and PR to exclude the possibility of most epithelial tumors and meningiomas; negative Langerin to exclude Langerhans cell-associated histiocytosis; negative CD21 to exclude out follicular dendritic cell sarcoma; negative GFAP and Oligo-2 to exclude glial tumors, such as glioblastoma and pleomorphic xanthoastrocytoma. For tumors with overlapping histological features, identifying molecular characteristics is crucial. For example: Both PCNSHS and high-grade gliomas are histologically characterized by pleomorphic tumor cells, abundant cytoplasm, significant mitotic activity, areas of necrosis, and spindle cells, which makes them difficult to distinguish (19). In addition, PCNSHS and glioma may also show overlapping molecular features, such as BRAF V600E mutation or BRAF gene fusion, CDKN2A/CDKN2B homozygous deletion, etc (20–22). However, gliomas typically do not express histiocytic markers and exhibit other molecular characteristics, including IDH mutations, ATRX alterations, 1p/19q co-deletions, EGFR amplification, TERT promoter mutations, H3 mutations, MYB/MYBL1 gene structural mutations, and FGFR1 mutations, etc (21). In conclusion, through a hierarchical strategy (first confirming the histiocytes origin, then systematically excluding tumors with overlapping morphological features, and finally verifying with molecular characteristics), the diagnostic specificity of PCNSHS can be improved.

It is well known that PCNSHS can manifest as suppurative inflammatory lesions and can therefore be easily confused with inflammatory lesions. This phenomenon can be repeatedly observed in numerous PCNSHS cases (3, 14, 15, 23). However, purulent material found during surgery is more likely a biological marker of active PCNSHS progression. Almefty et al. reported a case of PCNSHS in the left parietal lobe. The patient’s initial imaging and intraoperative findings showed purulent exudate, and pathology showed neutrophil infiltration and necrotizing inflammation. Recurrent purulent material post-surgery coexisted with heterotypic monocytic/multinucleated tumor cells in pathology. Despite repeated use of broad-spectrum antibiotics, purulent material and lesions reappeared (14). This suggests that the purulent material associated with PCNSHS is essentially secondary pathological changes accompanying tumor progression rather than an independent infection event. Clifton et al. also mentioned that this purulent substance may be a sign of disease progression (3). In our case, the initial post-operative pathology suggested inflammatory infiltration. The patient’s symptoms worsened only more than 2 months post-surgery, and pus along with an aggressive tumor was found during the second operation. It suggests that the persistence of inflammatory microenvironment may accelerate the recurrence process of PCNSHS. This may be due to chronic inflammation promoting tumor proliferation by releasing cytokines and angiogenesis factors, while the tumor itself induces secondary infection or inflammatory response, forming a vicious cycle.

Molecular diagnosis is often used as a supplement to tumor histology and pathological diagnosis. However, molecular diagnostic indicators for PCNSHS are limited and usually include routine tests such as T cell receptor gamma chain rearrangements, immunoglobulin heavy chain rearrangements, ALK alterations, and BRAF p.V600E mutations. The most common BRAF mutation in PCNSHS is BRAF p.V600E. However, Zhang et al. first reported a novel BRAF fusion variant called ARHGAP45::BRAF, found in pediatric PCNSHS (20). Marguet al. reported a case of multifocal PCNSHS. Molecular testing revealed extensive chromosomal abnormalities, particularly deletions of PTEN and CDKN2A genes, and emphasized that deletions of these genes may play a key role in the occurrence and progression of HS (24). May et al. reported a case of PCNSHS with a platelet-derived growth factor receptor (PDGFR) mutation and expression of PD-L1 and PD-L2, indicating the potential therapeutic effect of immune checkpoint inhibitors (9). Wang et al. reported a PCNSHS case with somatic NF2 mutations, enhancing our understanding of the molecular pathogenesis of this rare tumor (11). These findings suggest that the molecular characteristics and potential therapeutic targets of PCNSHS are complex and diverse, highlighting the need for further research to reveal its pathogenesis and explore more effective treatment strategies. Unfortunately, molecular testing was not performed in our case.

The etiology of PCNSHS is currently unclear and may be associated with a history of malignant hematological diseases and radiotherapy. One study reported a PCNSHS case with a prior history of chronic lymphoblastic leukemia (CLL). Due to limited sample materials, genetic rearrangement and further genetic testing could not be conducted, but clinical evidence strongly suggests that the PCNSHS may originate from previous CLL (25). Brown et al. reported on a 23-year-old male patient who developed PCNSHS seven years after remission of precursor B-cell acute lymphoblastic leukemia (B-ALL). Molecular testing revealed a clonal immunoglobulin heavy chain (IGH) gene rearrangement, suggesting that PCNSHS may have transformed from the patient’s prior B-ALL (26). Another study reported a 44-year-old male patient who developed PCNSHS 16 years after treatment for T-cell acute lymphoblastic leukemia (T-ALL) (27). These cases suggest that PCNSHS may be linked to prior hematological malignancies, with two potential theories proposed. One theory is that B-cell tumors and HS may originate from a common neoplastic progenitor cell, and B-cell tumors transform into HS through dedifferentiation and then redifferentiation. Another theory suggests that lymphoid tumors may transform into HS with different phenotypes but similar genotypes through direct transdifferentiation (28–31). In addition, a study reported a case of PCNSHS that occurred after radiotherapy. The authors established an etiological link between PCNSHS and prior radiotherapy, as well as its association with complex cytogenetic abnormalities in the tumors (8). Our patient had no previous history of lymphoma or other hematological malignancies and had not undergone tumor-related chemoradiotherapy.

PCNSHS is a highly aggressive malignant tumor with a poor prognosis. The median survival of patients is about 7 months, and the average survival is around 24 months (12). Although the optimal treatment plan for PCNSHS is unclear, surgery remains the primary treatment. For single lesions, surgery followed by postoperative radiotherapy is usually chosen; if the disease has turned multiple, more aggressive radiotherapy and combination chemotherapy are needed (9). Studies have shown that total resection is a key factor influencing overall survival and treatment outcomes in PCNSHS patients. Whether it is a single lesion or a solitary meningeal space-occupying lesion, complete resection of the tumor is closely associated with a better prognosis of the patient (2, 15, 32). Targeted therapy offers new hope for improving the prognosis of patients with PCNSHS. Vemurafenib is a targeted drug targeting the BRAF p.V600E mutation. Idbaih et al. reported a PCNSHS case with the BRAF p.V600E mutation. Within one month of receiving Vemurafenib treatment, the patient’s overall condition improved significantly. However, tumor recurrence and deterioration of neurological function ultimately led to the patient’s death (33). Another study reported a patient with PCNSHS with PDGFR mutation that was targeted by Dasatinib. However, the patient cannot tolerate Dasatinib due to side effects such as nausea, diarrhea and acute pancreatitis. In addition, the tumor also expressed PD-L1 and PD-L2, suggesting that immune checkpoint inhibitors may have potential therapeutic effects (9). In conclusion, although the treatment of PCNSHS still faces many challenges, the combination of surgery, chemoradiotherapy and targeted therapy offers a key treatment strategy to improve patient outcomes.

## Conclusion

This article reports a rare case of PCNSHS, and provides an in-depth discussion of previously reported cases of PCNSHS, focusing on analyzing the key points of its diagnosis and differential diagnosis. By integrating the clinical manifestations and pathological characteristics of this case, the diagnostic difficulties of PCNSHS were further clarified, and the importance of immunohistochemical markers in differential diagnosis was emphasized. In addition, the presence of purulent material and inflammatory lesions during surgery further complicates the diagnosis. Therefore, a comprehensive pathological analysis and differential diagnosis are essential for the accurate diagnosis of PCNSHS.

## Data Availability

The original contributions presented in the study are included in the article/supplementary material. Further inquiries can be directed to the corresponding author.
